# Pharmacological inhibition of RE1 silencing transcription factor disrupts SOX2 expression and neurogenesis in human induced pluripotent stem cells derived neuronal models

**DOI:** 10.1007/s11011-025-01744-1

**Published:** 2025-11-18

**Authors:** Ajmal Nassar, Sumukha Hegde, Divya Chandran, Suryadipali Pahadasingh, Aashika Nayak, Sairaj Satarker, Prasada Chowdari Gurram, Dinesh Upadhya, Madhavan Nampoothiri

**Affiliations:** 1 Department of Pharmacology, Manipal College of Pharmaceutical Sciences, Manipal Academy of Higher Education, Manipal, 576104 Karnataka India; 2 Centre for Molecular Neurosciences, Kasturba Medical College, Manipal Academy of Higher Education, Manipal, 576104 Karnataka India

**Keywords:** RE1-Silencing transcription factor (REST), Neurogenesis, SOX2, MAPK, WNT, Drug target

## Abstract

Background: The role of RE1-Silencing Transcription Factor (REST) in human neurogenesis remains unexplored. This study aims to investigate the expression patterns and possible targets of REST during the early stages of human neurogenesis. Methods: Human neural stem cells and neurons from human induced pluripotent stem cells (hiPSCs) were generated through established protocols. hiPSCs and derived neurons were characterized by immunocytochemistry (SOX2, OCT4, TUJ-1) and qPCR for pluripotency and neuronal marker expression. REST expression and the effect of REST inhibition using X5050 at different stages of development were evaluated using immunostaining. Further, proteomic analysis was performed to identify the key molecules and signalling pathways impacted by the inhibition of REST during neurogenesis. Western blotting and quantitative PCR were used to validate the key molecule targeted by REST inhibition. Results: Immunocytochemistry and qPCR examinations have affirmed the promising expression of pluripotency markers (SOX2, OCT4, Nanog) in hiPSCs and the neuronal marker TUJ-1 in differentiated neurons. REST expression is seen in hiPSCs, immature neurons, and mature neurons derived from hiPSCs. In immature neurons, REST proteins are seen in the soma and axons, while in mature neurons, REST is seen in the soma and is absent in the axons. The inhibition of REST in hiPSCs, NSCs and neuronal precursor cells with X5050 significantly reduced REST levels. Reduced REST levels in NSCs led to significant downregulation of essential proteins involved in neurogenesis, including SOX2, a key regulator of neural stem cell proliferation. REST inhibition by X5050 disrupted pivotal neurogenic signaling axis, including MAPK and WNT pathways, and reduced the mRNA expression of NESTIN, β-catenin (CTNNB1), and MAPK3, indicating perturbation of neural stem cell identity and key regulatory mechanisms. Conclusion: REST is a crucial regulator of human neurogenesis. REST is essential to drive neurogenesis as it controls the SOX2 levels during this stage. The role of REST in regulating neurogenic pathways offers novel perspectives on its potential as a target for therapy of neurodevelopmental diseases.

Graphical abstract

**Supplementary Information:**

The online version contains supplementary material available at 10.1007/s11011-025-01744-1.

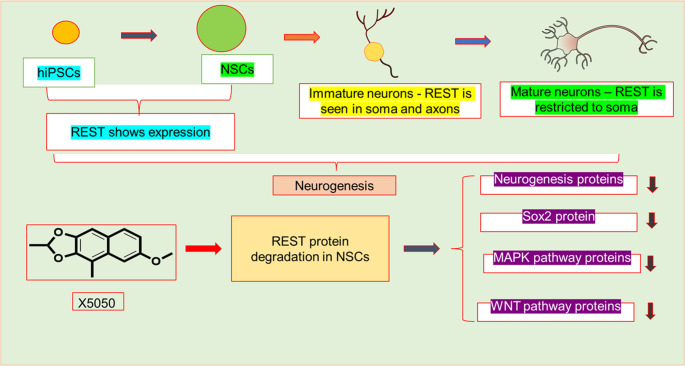

## Introduction

The RE-1 silencing transcription factor (REST), regularly referred to as neuron-restrictive silencing factor (NRSF), is a fundamental controller of neural gene transcription. (Chong et al. 1995; Schoenherr and Anderson 1995). The transcriptional repressor REST regulates neurogenesis by interacting with the highly conserved DNA sequence of its target genes, known as the RE-1 (Repressor Element 1) region, to prevent their expression. (Lunyak et al. 2002) REST has a predominant control over the genes influencing cellular differentiation.

REST is a neurogenesis inhibitor that interacts with target genes involved in brain differentiation and maturation (Gao et al. 2012) (Soldati et al. 2013). It fine-tunes neural gene expression and reduces neural gene expression in non-neuronal tissues (Ballas et al. 2005; Lunyak and Rosenfeld 2005; Aoki et al. 2012; Kok et al. 2012; Moravec et al. 2016). The activity of REST is significantly regulated by corepressors like CoREST and SIN3, as well as MECP2 and LSD1, along with several histone deacetylases (HDACs) (Huang et al. 1999; Battaglioli et al. 2002; Cunliffe 2008; Yang et al. 2018). REST has two isoforms, REST4 and REST1, with REST4 being the first to be identified (Tabuchi et al. 2002; Ovando-Roche et al. 2014). REST4 may prevent the repressive effects of REST. However, it has been observed that REST is downregulated while REST4 is activated with neural differentiation. The preservation and development of brain progenitor cells appear to depend on the balance between full-length REST and REST4 isoforms (Tabuchi et al. 2002; Ovando-Roche et al. 2014). More research has revealed that REST is essential later in life (Nassar et al. 2022), even though most studies have focused on mouse embryonic development. Animal studies have shown REST expression in postmitotic neurons in the late developmental stage and the postnatal brain (Palm et al. 1998; Calderone et al. 2003; Kuwabara et al. 2004; Sun et al. 2005). The presence of REST in regions such as the caudate nucleus, substantia nigra, hippocampus, and cortex disproves the idea that its level drops throughout differentiation (Prada et al. 2011). REST controls the development of the N-methyl-D-aspartate receptor (NMDAR) and K-Cl co-transporter (KCC2), the crucial synaptic and extrasynaptic proteins. REST binds to the Grin2b promoter, which codes for the NMDAR subunit GluN2B, silencing GluN2B expression. This encourages the transition of hippocampal synapses between the immature (mainly GluN2B-containing) and mature (mostly GluN2A-containing) NMDAR phenotype (Rodenas-Ruano et al. 2012). REST modulates proteins that control vesicle transport and release, axonal growth, and ion conductance, which are critical for synaptic plasticity and brain development (Sun et al. 2005; Kim et al. 2006; Greco et al. 2007; D’Alessandro et al. 2009; Gillies et al. 2009; Abrajano et al. 2009; Tan et al. 2010).

Despite the contradictory results of rodent studies, no research has demonstrated the crucial expression of REST in human neurons or provided any understanding of REST’s role during neurogenesis. The number of REST binding peaks in human embryonic stem cells (ESCs) is twice that of mouse ESCs, indicating a notable variation in REST cistromes between species. This implies that REST plays different regulatory roles in the genesis and biology of human and mouse stem cells. (Rockowitz and Zheng 2015). This study aims to investigate REST’s expression patterns and possible targets during the early stages of human neurogenesis by generating human neural stem cells and neurons from human induced pluripotent stem cells (hiPSCs). Expression of REST was identified in hiPSCs, neural stem cells (NSCs), immature and mature neurons. REST expression was inhibited using a pharmacological agent X5050 at different developmental stages. Previous work suggested that REST ablation can result in aberrant neurogenesis in Xenopus and chicken embryos, even though it supports neural differentiation and neurogenesis by derepressing neural genes. Since REST’s deactivation might upset the delicate equilibrium required for healthy neurogenesis, it appears that REST plays a more nuanced role in development. (Olguín et al. 2006) (Chen et al. 1998).

X5050 functions as a selective antagonist of REST, encouraging its proteasomal degradation to lessen REST-mediated transcriptional repression despite not altering REST’s transcriptional expression, RNA splicing, or DNA-binding affinity. In stem cell-derived neural paradigms from Huntington’s disease (HD) patients alongside in vivo murine models with striatal lesions, X5050 has been illustrated to restore neuronal gene expression by enhancing the expression of key neuronal genes, notably brain-derived neurotrophic factor (BDNF)(Charbord et al. 2013), (Zhao et al. 2017),(Lam et al. 2023). Although there are fewer publications on the REST inhibitor X5050, the available experimental findings underscore its potential as a focus of therapeutic intervention and support its use in experimental models to counteract aberrant REST activity and enhance neuronal function in neurodegenerative and other neuropathological disorders.

Thus, in this study, key molecules impacted by the inhibition of REST during neurogenesis, X5050-treated NSCs, were evaluated for proteomic analysis and validated through western blot. Key neurogenesis-related proteins and signaling pathways were assessed to elucidate the impact of REST suppression. A crucial transcription factor for preserving the identity of neural progenitors and directing neurodevelopmental processes is SOX2. There may be a regulatory connection between SOX2 expression and REST, a well-known transcriptional repressor.

REST essentially orchestrates human neurogenesis, whose activity is essential for maintaining SOX2 expression and correctly regulating important neurogenic cascades like MAPK and WNT. Pharmacologically inhibiting REST by X5050 disrupts these processes, ultimately compromising neuronal ontogeny.

## Materials and methods

### Culture and maintenance of HiPSCs

hiPSCs were cultured on a matrigel-coated plate (Corning, Cat. No. 354277) in StemFlex Medium (Thermo Fisher Scientific, Cat. No. A3349401) at 37 °C with 5% CO₂ as described in our earlier paper (RAB et al. 2024). Using PSC Neural Induction Medium (Thermo Fisher Scientific, Cat. No. A1647801), neuroepithelial commitment was established on day three and maintained for another 8 days. By day 9, neural stem cells were characterized. Once characterized, NSCs were cultured in neural maintenance medium and treated with 10 µM REST inhibitor at ~ 60% confluence. Cells were incubated with the inhibitor for different time intervals, after which they were dissociated using dispase and used for various studies.

### Quantitative PCR

Total RNA was isolated using the Trizol–chloroform method, and RNA concentration was quantified with a Qubit fluorometer. For cDNA synthesis, 250 ng RNA from both control and treated samples was reverse transcribed using the PrimeScript™ cDNA Synthesis Kit (Takara, Cat. No. 6110B). RT-qPCR was performed on the Insta Q96 Real-Time PCR system. Primers were initially tested on a gradient PCR to optimize annealing temperature; an annealing temperature of 57 °C and a melting temperature of 59 °C provided optimal amplification and melt curves. All reactions were run in triplicates for both control and treated groups. ΔCt and ΔΔCt values were calculated, and the relative fold change in gene expression was determined.

### Immunofluorescence staining

Cells were fixed in 4% paraformaldehyde (PFA; Sigma, Cat. No. P6148) for 10 min, permeabilized for 5 min with 0.2% Triton X-100 (Sigma, Cat. No. X100) and then incubated for 1 h in a blocking solution containing 3% bovine serum albumin (BSA; Thermo, Cat. No. BP1600-100) supplemented with 0.2% Triton X-100. The following primary antibodies were used to incubate overnight at 4 °C with the cells. SOX2 (Abcam, Cat. No. ab97959; RRID: AB_2341193), REST (Abcam, Cat. No. ab315007; RRID: AB_10585428), OCT4 (Abcam, Cat. No. ab109183; RRID: AB_10864777), and TUJ1 (Abcam, Cat. No. ab7751; RRID: AB_306045). Following three washes, cells were incubated with Alexa Fluor-488 and 568 (Thermo Fisher Scientific, Cat. Nos. A-11001, A-11032) for an hour. Following counterstaining with DAPI (Roche, Cat. No. 10236276001), cells were observed and photographed using the Olympus BX53 epifluorescence microscope. Experiments were performed using at least three independent biological replicates from separate hiPSC cultures.

### Western blotting

The hiPSCs (day 1) and NSCs (days 1 and 9) were subjected to Western blotting with or without X5050 exposure. TrypLE Express (Thermo Fisher Scientific, Cat. No. 12605010) was used to enzymatically dissociate the cells after propagating to roughly 70% confluency in 6-well culture vessels. The cells were centrifuged at 10,000 rpm for three to five minutes at 4 °C. Dulbecco’s phosphate-buffered saline (DPBS) has been employed to rinse the resultant cellular pellets, and RIPA buffer (Sigma) enhanced with Halt™ Protease Inhibitor Cocktail (Thermo Fisher Scientific, Cat. No. 78429 or 87785) was used to lyse them.

The PierceTM BCA Protein Assay Kit (Thermo Fisher Scientific, Cat. No. 23225) was used to quantify the protein concentration. After separating protein aliquots on 10% SDS-polyacrylamide gels (Genescript), they were transferred onto PVDF membranes and were blocked employing primary antibodies against REST (Abcam, Cat. No. ab315007; RRID: AB_10585428) and SOX2 (Abcam, Cat. No. ab97959; RRID: AB_2341193) in 5% non-fat dried milk dissolved in TBST and incubated for the entire night at 4 °C. After 16 h, membranes were washed and treated with a secondary antibody conjugated with horseradish peroxidase (HRP) (Thermo Fisher Scientific, Cat. No. 32430). The SuperSignalTM West Pico PLUS Chemiluminescent Substrate (Thermo Fisher Scientific, Cat. No. 34578) was used to detect the signal. Membranes were stripped and reprobed for β-actin. Experiments were performed using at least three independent biological replicates from separate hiPSC cultures.

### Proteomic analysis

We pooled NSCs treated with and without X5050 samples from duplicate experiments for proteomic analysis. Proteins were separated and digested into peptides, reconstituted in 0.1% formic acid solution, and underwent LC-MS analysis using a commercial C18 column (Acclaim PepMap RSLC, 75 μm × 50 cm, Thermo Scientific) and an Orbitrap Fusion Tribrid mass spectrometer. The resultant MS/MS spectra were compared to the UniProt Human database (https://www.uniprot.org) using the SEQUEST search engine within the Proteome Discoverer 2.4 software (Thermo Scientific). For label-free quantification, proteins validated by two or more peptide matches were considered. Protein ratios were computed using the median of all peptide hits linked to each protein. The variability in these ratios was used to indicate quantitative precision.

### Statistical analysis

GraphPad Prism (v 7.0.5) was used for statistical analysis. Results were expressed as mean ± SEM. Unpaired t-tests were employed for comparison of results between 2 groups. One-way ANOVA was used to analyze when three or more groups were compared. *p* < 0.05 was considered statistically significant. Western blot data were analysed using ImageJ and normalized to β-actin. In proteome analysis, DEPs (differentially expressed proteins) were defined as those with *p* < 0.05 and fold change > 1 or <– 1. DEPs were visualized using Venn diagrams and volcano plots, with significance thresholds set at *p* < 0.001, *p* < 0.01, and *p* < 0.05. Three independent biological replicates, each analyzed in technical triplicate (*n* = 3 biological × 3 technical replicates) were used for quantitative PCR.

## Results

### Molecular validation of HiPSCs and hiPSC-derived neurons

**Fig. 1:**
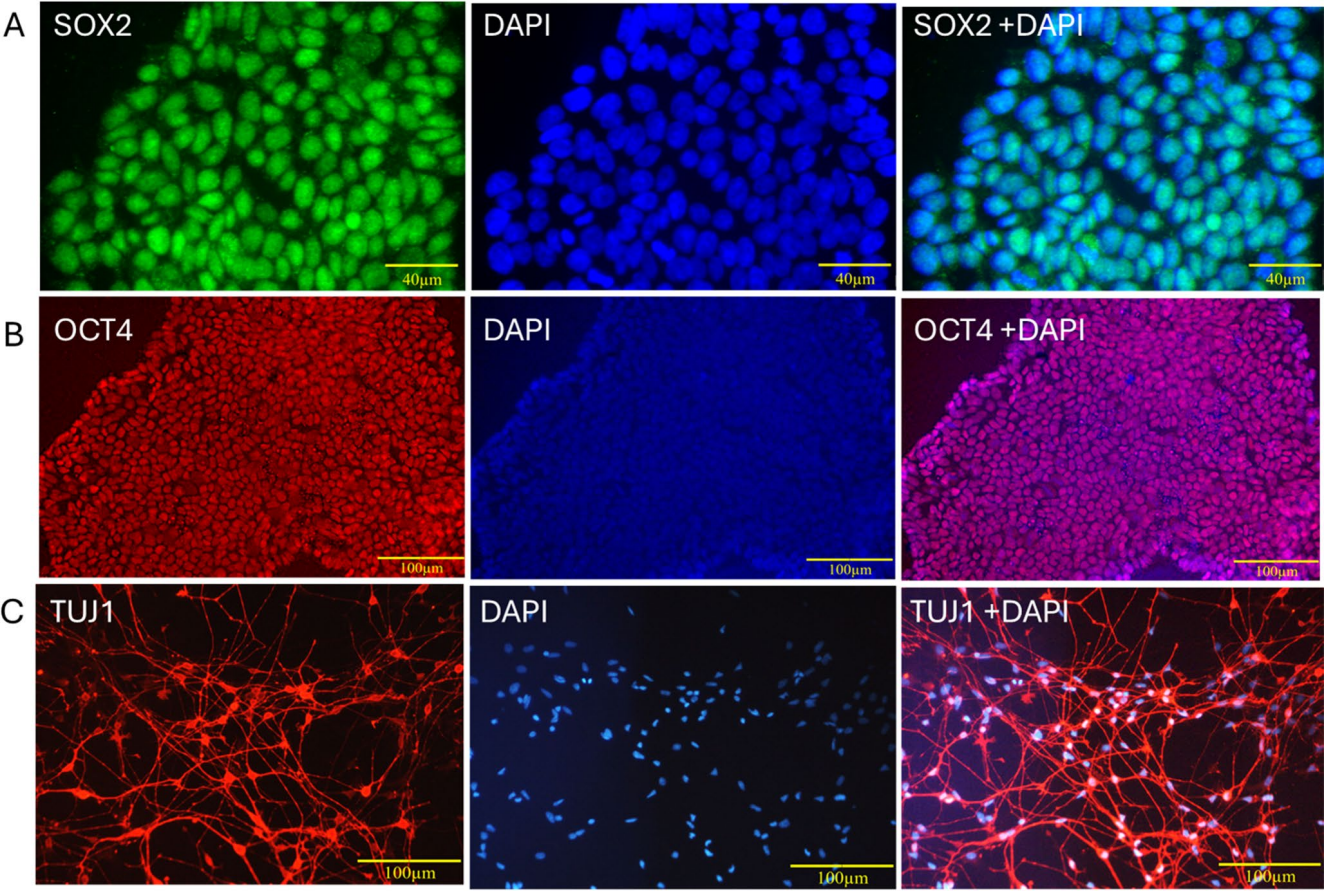
Characterization of Human iPSCs by Pluripotency Markers (SOX2, OCT4) and Differentiation into Neurons Expressing TUJ-1. Panels A and B demonstrate hiPSC characterization (day 2 of culture), while panel C demonstrates neuronal characterization at day 21 of neuronal differentiation. Panel A shows SOX2-positive cells counterstained with DAPI and merged images of SOX2 with DAPI. Panel B shows OCT4-positive cells counterstained with DAPI and merged images of OCT4 with DAPI. Panel C shows TUJ-1-positive cells counterstained with DAPI and merged images of TUJ-1 with DAPI. Scale bar = 50 μm. Characterization of hiPSCs and hiPSC-derived neurons

**Fig. 2:**
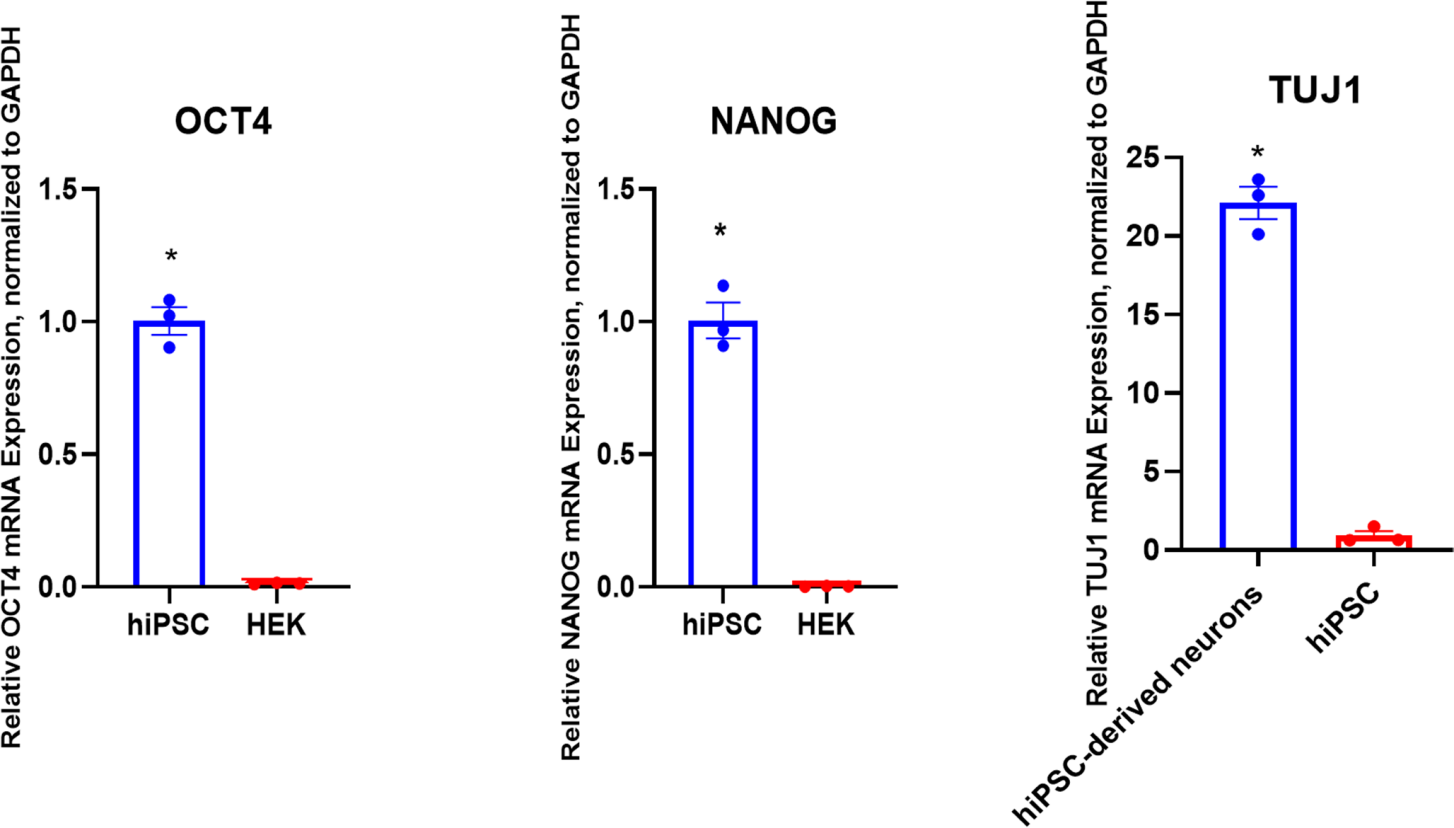
Characterization of hiPSCs (day 2) and hiPSC-derived neurons (day 21) through qPCR validation. **A**. Demonstrates a high amount of OCT 4 expression in the hiPSCs compared to HEK 293 cells. **B **demonstrates higher levels of Nanog expression in the hiPSCs than HEK 293 cells. **C**. Demonstrates higher levels of TUJ-1 expression in the neurons compared to hiPSCs. Data represent mean ± SEM of three independent biological replicates, each analyzed in technical triplicate (*n* = 3 biological × 3 technical replicates). Statistical significance was determined using an unpaired t-test. Characterization of hiPSCs and hiPSC-derived neurons through qPCR validation

The pluripotent state of hiPSCs was validated on day 2 of culture through SOX2 and OCT4 immunoreactivity, whereas neuronal lineage commitment was confirmed on day 21 of neuronal culture through TUJ-1 expression. Figure 1A and B showcase strong nuclear expression of SOX2 along with OCT4 in hiPSCs. Nuclear localization was confirmed by DAPI counterstaining, and the colocalization of SOX2 and OCT4 with DAPI was discernible in the merged images. Additionally, we employed the qPCR approach to verify the specific identity of the generated hiPSCs. We included HEK293 cells, which do not express stem cell markers, as a negative control for comparison (Fig. 2). The pluripotency genes OCT4 (Fig. 2A) and Nanog (Fig. 2B) were significantly higher in hiPSCs than in HEK293 cells, confirming their undifferentiated state. Further, we generated neurons derived from iPSCs, which exhibited prominent expression of the early neuronal marker TUJ1 (βIII-tubulin) during immunostaining, signifying efficient differentiation (Fig. 1C). Moreover, we confirmed that TUJ1 expression is significantly higher in mature iPSC-derived neurons compared to HEK293 cells, as validated by qPCR. Furthermore, in contrast to undifferentiated hiPSCs, we found that TUJ1 expression was substantially higher in hiPSC-derived neurons (Fig. 2C), confirming effective lineage commitment toward the neuronal fate.

### The hiPSCs, immature and mature neurons derived from hiPSCs, demonstrate REST expression

**Fig. 3:**
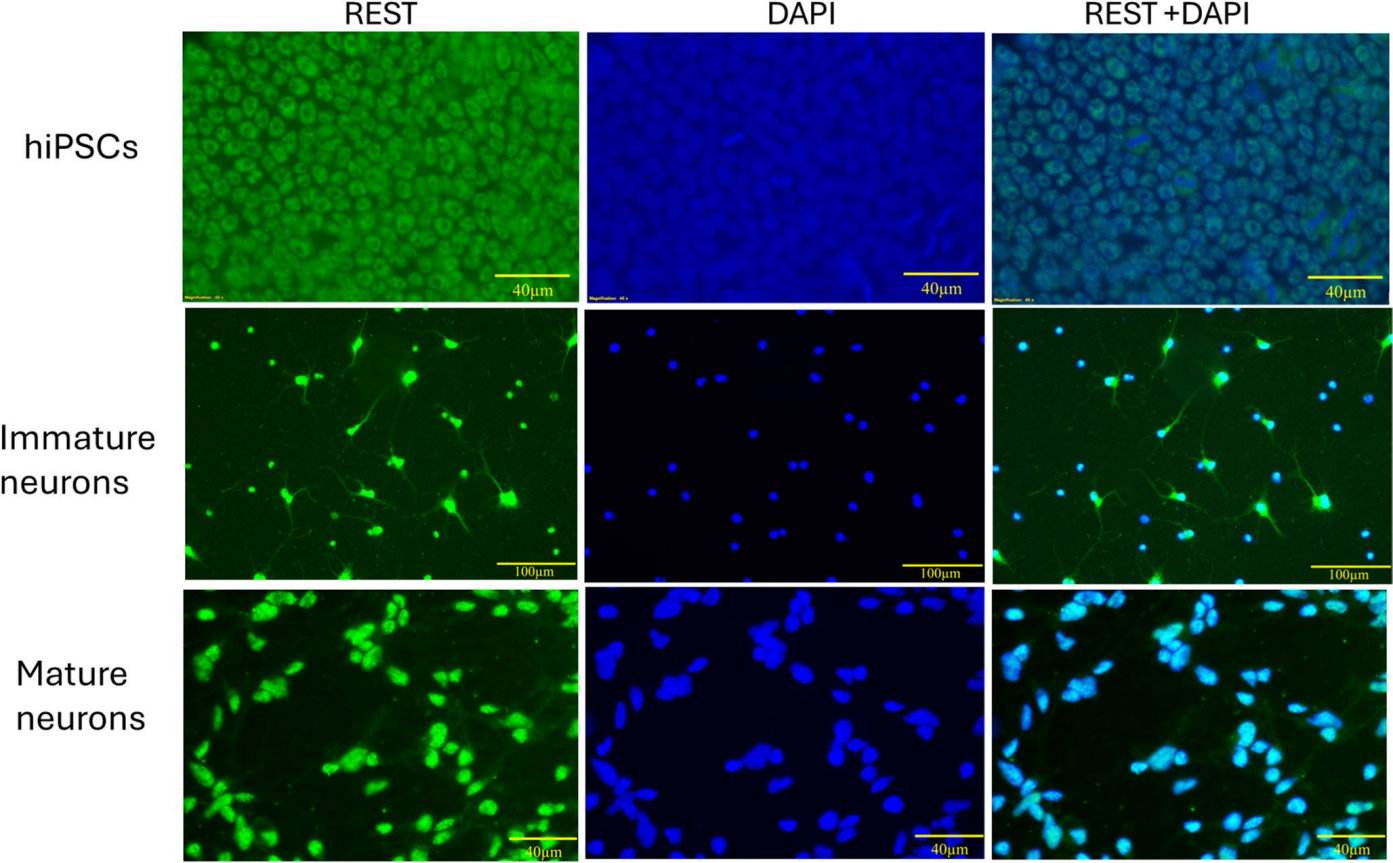
REST Expression in hiPSCs and hiPSC-Derived Neurons

An analysis of REST expression at different stages of neurogenesis, including hiPSCs and hiPSC-derived immature and mature neurons, demonstrated through immunostaining that REST is present consistently across all these phases of neuronal development. At the stem cell stage, REST levels were observed in the cytoplasm. In contrast, in both immature and mature neurons, REST was primarily located inside the nucleus following differentiation and was notably absent from the axonal region (Indicated in Fig. 3). In immature neurons, REST proteins are seen in the soma and axons, while in mature neurons, REST is seen in the soma but absent in the axons.

REST expression (green) and nuclear staining (DAPI, blue) in (a) hiPSCs (day 2), (b) hiPSC-derived immature neurons (day 10 neurons), and (c) hiPSC-derived mature neurons demonstrate expression only in the soma (Day 30 neurons). Images are representative of three independent biological replicates. Scale bar = 50 μm.

### X5050 decreases REST protein levels in a time-dependent manner

**Fig. 4:**
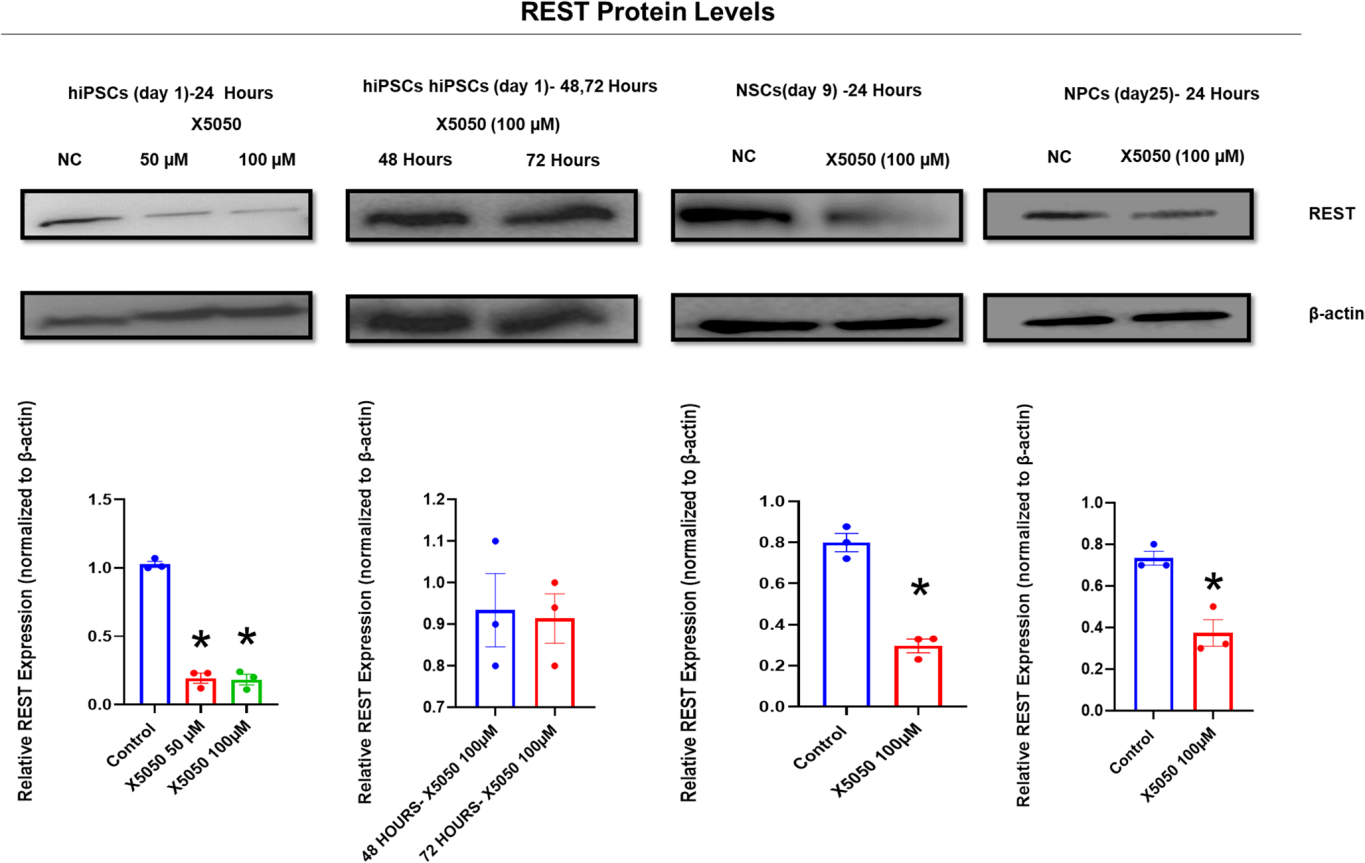
REST expression following X5050 treatment in human induced pluripotent stem cells

Western blotting was used to examine the effectiveness of the REST inhibitor, X5050, in degrading the REST protein at various dosages and exposure durations. REST protein levels in hiPSCs (day 1) were decreased at 50 and 100 µM concentrations for up to 24 h, respectively, whereas in 100 µM-treated hiPSCs, protein levels recovered after 48 and 72 h. It suggests that X5050 decreases REST protein levels in a time-dependent way, with the capacity to degrade REST for only 24 h. Additionally, we used X5050 at 100 µM for 24 h to perform REST inhibition in NSCs (day 4)- 24 h and NSCs exposed to X5050 (50 µM/day) till 9 days and days 25 neural progenitors. We have also discovered considerable REST inhibition in these cells. (Indicated in Fig. 4)

Western blot analysis of REST and β-actin in (a) hiPSCs (day 1) treated with X5050 at 50 µM and 100 µM for 24 h, (b) hiPSCs treated with X5050 at 100 µM for 48 and 72 h, (c) NSCs (day 4) treated with X5050 at 100 µM for 24 h, and (d) neural progenitor cells (Day 25) treated with X5050 at 100 µM for 24 h. Data represent mean ± SEM of three independent biological replicates. Statistical significance was determined using one-way ANOVA followed by Tukey’s post-hoc test for 3 groups and an unpaired t-test for 2 groups.

### Proteomic analysis of REST-deficient NSCs and the control group

#### X5050 causes significant alterations in the proteins that control neurogenesis and stem cell proliferation

**Fig. 5:**
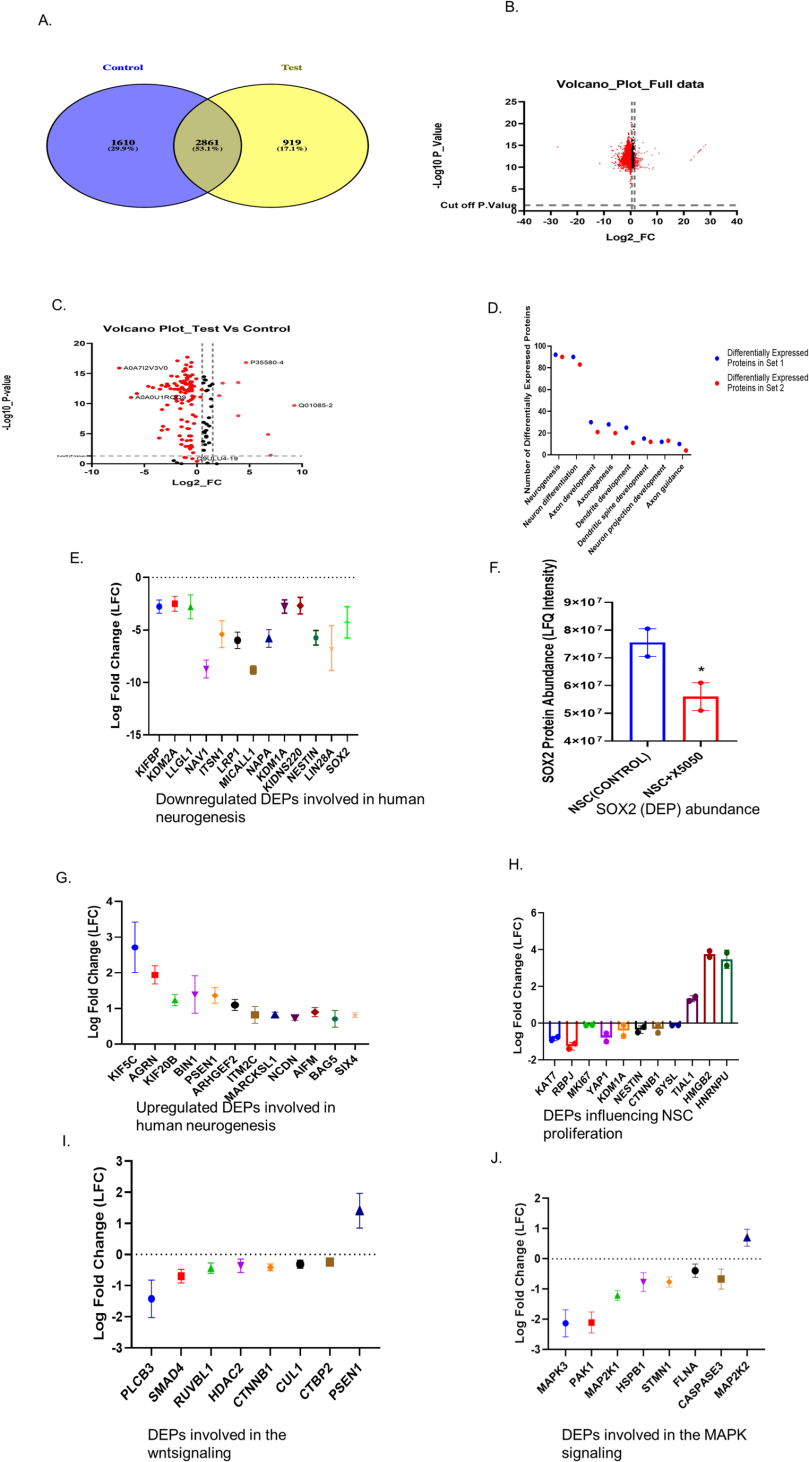
Integrated visualization of differentially expressed proteins (DEPs) and pathway analyses in NSCs (day 3) treated with X5050 (100 µM). (A) Venn diagram showing distinct protein expression patterns in NSCs following X5050 treatment. (B) Volcano plot of the total dataset illustrating overall differential protein expression; the x-axis represents log₂ fold change (upregulation or downregulation), and the y-axis indicates statistical significance as –log₁₀(p-value). (C) Volcano plot focused on neurogenesis-related DEPs, highlighting significantly upregulated and downregulated proteins (*p* < 0.05, fold change > 1 or < 1). (D) Bar graph showing the number of DEPs associated with neurogenesis and related Gene Ontology (GO) biological processes in X5050-treated NSCs. (E) Downregulated DEPs involved in human neurogenesis based on log₂ fold change values (GO-derived). (F) Comparison of SOX2 abundance between control and X5050-treated NSCs, indicating its significant downregulation. (G) Upregulated DEPs involved in human neurogenesis, based on GO analysis and log₂ fold change values. (H) Combined representation of both upregulated and downregulated DEPs influencing NSC proliferation. (I) GO and KEGG pathway analysis highlighting top DEPs in the WNT signaling pathway. (J) GO and KEGG pathway analysis highlighting top DEPs in the MAPK signaling pathway. Two independent biological replicates per condition were analyzed, with each replicate measured in technical duplicate. Technical replicates were averaged, and statistical comparisons between the two groups were made using an unpaired t-test

Comparative proteomic quantification of NSCs treated with and without X5050 was conducted to ascertain the impact of REST absence on NSCs. We used pooled samples for the proteomics experiment. Following data filtering and analysis, 1610 proteins were found of which 919 proteins displayed significant change (Shown in Fig. 5.A). Among these 919 differentially expressed proteins (DEPs), 80% were downregulated in NSCs upon REST inhibition. (Shown in Fig. 5.B). According to the gene ontology study, the most striking declines were found in proteins involved in neurogenesis and stem cell proliferation. 92 DEPs that have an impact on neurogenesis were downregulated when REST was inhibited. (Shown in Fig. 5.C). 92 proteins that are actively involved in numerous crucial neurogenesis proteins, including proteins facilitating neural differentiation, axon development, and dendritic development, were inhibited by X5050 treatment. (Shown in Fig. 5.D). Similarly, the main increases were detected in proteins involved in the negative regulation of the neurogenesis class. It was found that X5050 significantly reduced the expression of the proteins Lin28a, SOX2, NES, and CTBP2, all of which positively regulate neurogenesis (Zhang 2014; Nowak et al. 2014; Bernal and Arranz 2018; Karaca et al. 2020). (Indicated in Fig. 5. E and F). The proteins that inhibit neurogenesis, such as BAG5, PSEN1, and SPAG9 (not shown), were elevated during REST inhibition (Kilb et al. 2004; Xu et al. 2010). Furthermore, blocking REST resulted in the elevation of 12 DEPs that were encouraging the formation of NSCs (Indicated in Fig. 5.G). Stem cell proliferation genes study (GO:0072089) revealed that several genes, including BYSL, KAT7, RBPJ, MKI67, YAP1, KDM1A, XRCC5, NES, EIF2AK2, and CTNNB1, were downregulated, whereas HNRNPU, HMGB2, and TIAL1 were found to be elevated (Indicated in Fig. 5.H). It was observed that seven DEPs that were actively involved in stem cell proliferation were inhibited upon loss of REST, including MKI67, a critical marker of stem cell proliferation, which was found to be decreased upon loss of REST.

### REST inhibition causes significant alterations of the proteins that impact the MAPK signaling cascade and the WNT signaling pathway

The major WNT signaling influencing proteins were identified based on information from the Kegg pathway entry HSA04310 and the ontology GO:0016055. We found that X5050 decreased the expression of proteins influencing the WNT signaling pathway, such as CTNNB1, CTBP2, PLCB3, SKP1, CUL1, RUVBL1, HDAC2, and SMAD4. (Indicated in Fig. 5.I). It was also demonstrated that X5050 caused an increase in the PSEN1 gene. Presenilin1 (PSEN1) was present in human NSCs, and it has been speculated that PSEN1 may promote CTNNB1 breakdown in embryonic stem cells (Yang et al. 2020). Our findings are corroborated by a published study by Lue et al., which demonstrated that REST depletion enhances PSEN1 in AD (Lu et al. 2014). It implies that PSEN1 is a particular REST target in both NSCs and AD. (Indicated in Fig. 5.I). Further key MAP signaling cascade regulators were identified by gene ontology (ID 0000165) and KEGG pathway (ID map04010). MAPK3, MAP2K1, FLNA, HSPB1, PAK1, STMN1, CASP3 were all significantly downregulated. Interestingly, MAP2K2 appears to be upregulated, suggesting that the REST may regulate MAP2K2 differently (Shown in Fig. 5.J).

Nishihara et al. were the first to put forward the association between REST and WNT signaling, and they illustrated that both Cyclin D1 and REST are directly influenced by canonical WNT signaling in chick neural progenitor cells (Nishihara et al. 2003). On the other hand, a different group of scientists has shown that REST knockdown led to an increase in MAPK and WNT signaling during differentiation (Thakore-Shah et al. 2015). Even though a few recent studies have focused on the impact of WNT signaling and REST expression in various disorders that affect aging, such as HD (De Souza et al. 2020) and AD (Lu et al. 2014) the strong relationship between REST and WNT signaling has been proven. According to string analysis, REST loss may impact the NSC’s sustaining circuit by directly inhibiting SOX2 or altering HDAC2 and STMN1, which affect SOX2. The important proteins that control the MAPK and WNT signaling pathways, including MAPK3, STMN1, CASP3, CTNNB1, HDAC2, CTBP2, and HSPB1, may also be disrupted if the REST-SOX2 circuit is interrupted.

### Validation and support of proteomic findings

#### Expression levels of REST and SOX2 during neuronal development

**Fig. 6:**
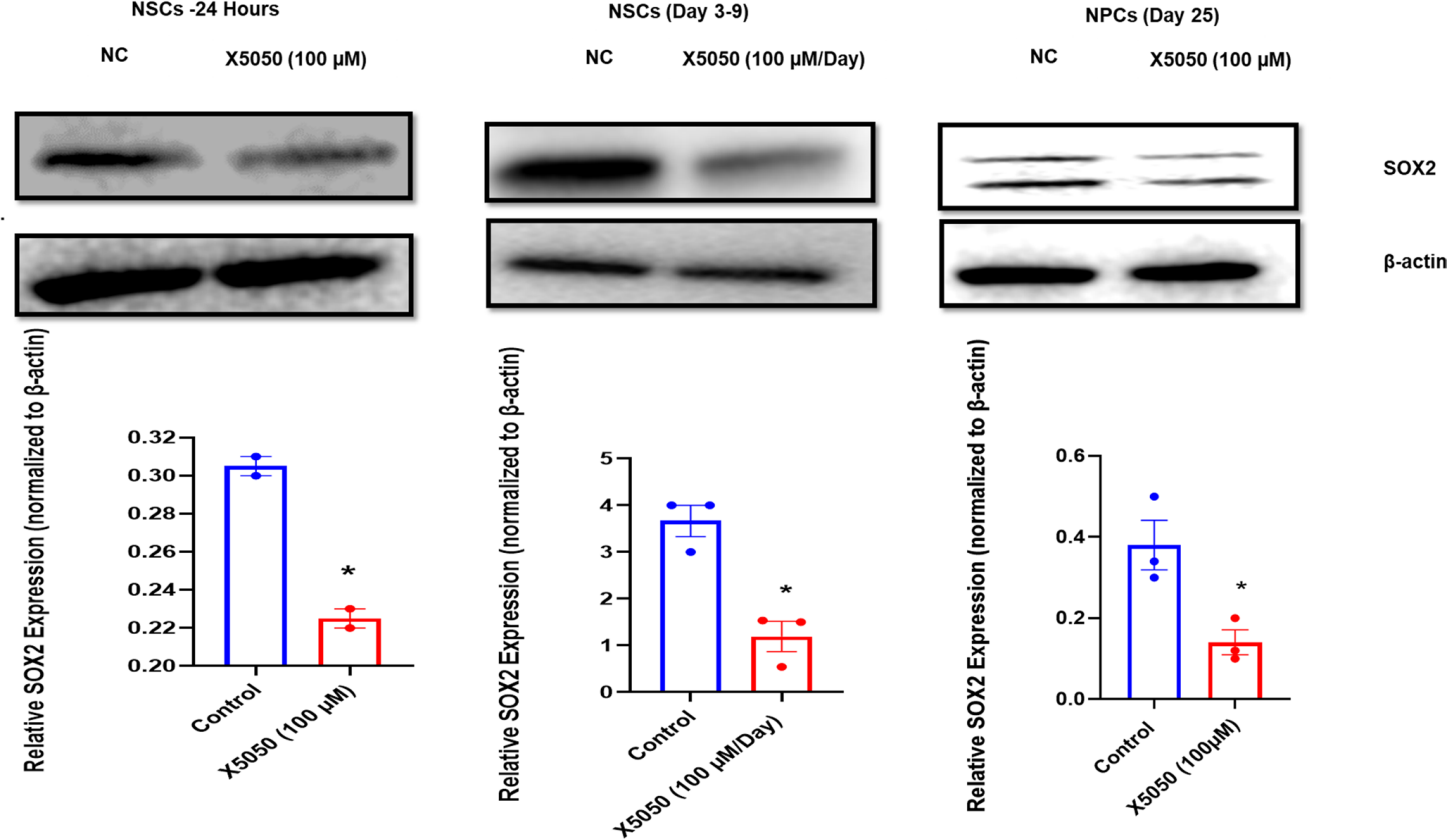
SOX2 expression in NSCs and NPCs following REST Inhibition

The study of the proteomic data revealed that one of the important genes that regulates neural differentiation, SOX2, is downregulated in response to REST inhibition by X5050. Western blotting was done in NSCs (day 4), 24 h, NSCs-9 days, and NPCs (day 25) with and without REST inhibition to validate the relationship between REST and SOX2. Interestingly, SOX2 expression decreased in all the above-mentioned cell types following REST inhibition (Fig. 6), suggesting a functional interplay between REST and SOX2 in regulating gene expression during brain development, although direct physical interaction between the two proteins has not been established. The primary limitation of this investigation is that only SOX2 had been confirmed by Western blot, while critical candidates from the MAPK and WNT cascades revealed in the proteomic survey remain unsubstantiated and should be verified in subsequent studies.

Western blot analysis of SOX2 and β-actin in (a) NSCs treated with X5050 (100 µM) for 24 h, (b) NSCs treated with X5050 (100 µM) every 24 h from Day 3 to Day 9, with Western blotting performed on Day 9, and (c) NPCs at Day 25 with and without X5050 treatment. The results demonstrate decreased SOX2 levels in all conditions with REST inhibition. Data represent mean ± SEM of three independent biological replicates. Statistical significance was determined using one-way ANOVA followed by Tukey’s post-hoc test for 3 groups and an unpaired t-test for 2 groups )

### Transcript-Level support for proteomic observations

**Fig. 7:**
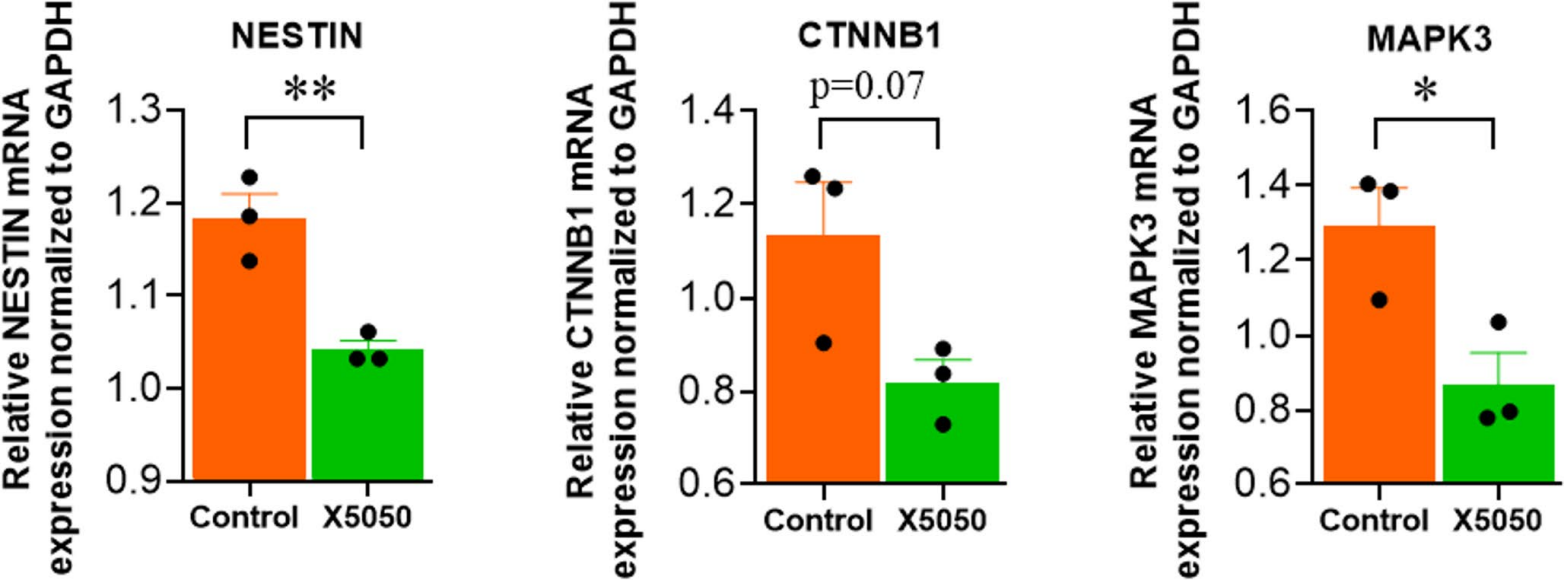
Quantitative PCR Analysis of Gene Expression in NSCs Treated with X5050 (100 μM)

Western blotting substantiated the proteomics-identified diminution of SOX2 protein. In parallel, quantitative PCR revealed uniform attenuation of NESTIN, CTNNB1, and MAPK3 transcripts upon X5050 (100 µM) treatment of NSCs, furnishing supportive concordance with the proteomic data(Fig. 7), . While not a surrogate for protein validation, these transcriptomic alterations provide corroborative evidence, warranting future confirmation through targeted proteomic approaches.

Quantitative PCR analysis of Nestin, β-catenin, and MAPK3 mRNA levels in neural stem cells (NSCs) treated with X5050 (100 µM) for 24 h. Expression values were normalized to GAPDH. All three genes showed significant downregulation relative to vehicle controls. Data represent mean ± SEM of three independent biological replicates, each measured in technical triplicate. Statistical significance was determined using an unpaired t-test.

## Discussion

Human neurogenesis has been controlled to guarantee the generation of fully functional neurons by the regulated activity of numerous epigenetic repressors (Jakovcevski and Akbarian 2012). It could be distinguished between the effects of genetic and non-genetic factors on the development of human neuronal circuitry owing to the newly discovered use of hiPSCs, which can differentiate into any neurons and mimic the course of early brain development while retaining the unique genetic signature of humans (Ardhanareeswaran et al. 2017). Transcription factors (TFs) represent a noteworthy category of developmental regulators. Numerous TFs function as decision-makers by controlling the timing of gene expression, which in turn controls the order in which the phases of differentiation occur (Lee et al. 2014). REST transcription is markedly suppressed at the end of neural differentiation. Progenitors may be unable to differentiate into neurons if REST expression persists correctly (Du et al. 2018). REST is steadily downregulated to permit the emergence of the adult neuronal phenotype. This drop must occur to relieve the repression of genes vital to neuronal differentiation, allowing progenitor cells to activate the gene networks crucial for the specialized functions and characteristics of the nervous system (Ballas et al. 2005) (Lunyak and Rosenfeld 2005; Nechiporuk et al. 2016). Still, it is an open question whether the targets of REST and the time at which REST and REST targets influence the maturation of neural phenotype. This study aimed to conduct a molecular-level investigation of human neurogenesis by analyzing REST location and its level during the various stages of differentiation. Rodent models demonstrated that it has a critical role in neurogenesis.

To understand how REST regulates the process of human neurogenesis, we used a novel REST inhibitor, X5050. X5050, as a REST inhibitor, was developed by a group of researchers in 2013. X5050 reduces REST in Human embryonic kidney (HEK293) cells in a dose-responsive manner and is cellularly permeable. It is believed that X5050 is intended to affect REST degradation instead of its expression or binding to the RE1 sequence. In rats with striatal injuries caused by quinolinic acid, X5050 was demonstrated to restore REST-regulated gene expressions, including BDNF in the prefrontal cortex. Additionally, it has been shown that REST decline facilitates BDNF and SNAP25 elevation in human brain stem cells. (Charbord et al. 2013).

At both 50 and 100 µM concentrations, the REST inhibitor X5050 decreases REST protein levels for 24 h in hiPSCs, although protein levels rise after 48 and 72 h in hiPSCs treated with X5050 at 100 µM. It suggests that X5050 decreases REST protein levels in a time-dependent manner. No fall in REST level has been seen after treatment with X5050 in the hiPSCs for 48 and 72 h. X5050 can degrade the REST protein without impacting its mRNA levels (Charbord et al. 2013), which reveals that it may restrict REST activity for up to 24 h by diminishing its protein levels. The possible reasons for no observed REST inhibition in 48 and 72 h might be sustained mRNA transcription machinery, which continues to run smoothly, and REST production continues as usual. The observed increase in REST levels after 24 h may also be attributed to the degradation of X5050 over time, resulting in diminished inhibition of REST.

REST was found inside the cells, according to our substantiated results. According to immunofluorescence results, REST was present inside the hiPSCs, primarily in the cytoplasm and lacking in the nucleus. However, we observed the REST level within the nucleus as the hiPSCs evolved into immature and mature neurons. In immature neurons, REST proteins are seen in the soma and axons, while in mature neurons, REST is seen in the soma and is absent in the axons. Our results could provide insights into the nuclear translocation of REST from the stem cell stage through differentiation. However, further experiments are needed to validate this process and better understand REST’s role in neuronal development. The observation that REST tends to remain in the nucleus during differentiation is critical. The shuttle of REST from the cytoplasm to the nucleus has been observed. There have also been reports of the bidirectional translocation of REST between the nucleus and cytoplasm in neurodegenerative disorders, including AD, PD, and HD. REST was recently found to be present in the nucleus rather than the cytoplasm in PD and AD circumstances, and it is this diminished nuclear availability of REST drives AD and PD progression. On the other hand, HD progression begins with REST entry into the nucleus (Nassar et al. 2023, 2024). A constraint of our investigation is that the inference regarding REST disposition—cytoplasmic in hiPSCs and nuclear in neurons—derives solely from immunocytochemical visualization. While this modality affords suggestive evidence, it does not furnish the biochemical rigor that nuclear–cytoplasmic fractionation would confer. Prospective inquiries employing fractionation or allied approaches will be required to substantiate these observations.

According to proteomic results, REST loss significantly influenced many neurogenesis-related proteins. The potential of REST to influence multiple genes involved in brain development, primarily SOX2, was validated and correlated with the outcomes of proteomic analysis and western blotting. As a whole, proteomic outcomes revealed certain differentially reflected proteins that uncovered peculiar molecular distinctions among REST-inhibited cells and normal cells, indirectly indicating putative biological processes associated with neurogenesis and its crucial aspects, including axon guidance, neural projection development, and dendritic development. In addition, several stem cell proliferation markers showed considerable downregulation upon REST inhibition, suggesting that REST inhibition impacts NSCs’ capacity for proliferation. Several genes, including BYSL, KAT7, MKI67, YAP1, KDM1A, NES, and CTNNB1, were downregulated, whereas HNRNPU, HMGB2, and TIAL1 were found to be elevated. Although it was discovered that the majority of the positively regulating genes influencing stem cell proliferation were downregulated upon REST inhibition, it was also discovered that X5050 treatment has an impact on some positively regulating genes for cell proliferation, including HNRNPU, HMGB2, and TIAL1. It suggests that REST inhibition primarily affects stem cell proliferation, but it also has the power to modify some genes that favorably influence cell proliferation. Many studies have conveyed that REST acts as a negative regulator of differentiation (Ballas et al. 2005; Mukherjee et al. 2016). Furthermore, research studies have shown that REST is diminished during neural development, and it has also been shown that the cAMP-elevating agents forskolin and IBMX facilitate MSCs to differentiate in a way that is similar to that of neurons by reducing REST and then inducing the neural phenotype (Thompson et al. 2019). However, a contradictory study also reported REST as a positive regulator of neural differentiation in developing mouse brains (Raj et al. 2011). Like the results of this study, our research also pinpointed REST as a beneficial regulator of neural differentiation. Protein quantification of proteins in REST-inhibited human NSCs highlighted the critical regulation of REST during neurogenesis. Diminished levels of SOX2 were observed in REST-inhibited cells. The expression of SOX2 has implications for neuronal differentiation and is a prerequisite for conserving the characteristics of neural progenitor stem cells during differentiation. Proper transcriptional and post-translational mechanisms guarantee the right amount of SOX2 for normal brain development (Sikorska et al. 2008; Andreu-Agullo et al. 2012; Julian et al. 2013; Cui et al. 2018). NSCs with the SOX2 mutation cannot turn into neurons (Mercurio et al. 2022). Previous research has demonstrated a conflicting relationship between REST and SOX2. Mouse embryonic stem cells displayed decreased SOX2 expression during REST inhibition (Singh et al. 2008) but not in human embryonic stem cells, which had no effect (Thakore-Shah et al. 2015). These outcomes reveal that mouse and human embryonic stem cells, REST and SOX2, may have distinct purposes. Most importantly, we found that human brain stem cells and progenitors had lower SOX2 levels when REST was inhibited. According to this, REST governs SOX2, and a drop in REST has an immense effect on SOX2 expression, eventually leading to the dysregulation of REST target genes. Quantitative PCR analysis confirmed that NESTIN and MAPK3 were significantly downregulated following X5050 treatment. At the same time, β-catenin (CTNNB1) was non-significantly reduced, supporting the proteomic findings and suggesting that REST inhibition may impact neural stem cell identity and key WNT/MAPK signaling pathways. Further protein-level validation will be necessary to establish these effects fully.

Overall, our outcomes confirm that REST loss has an enormous consequence for human neurogenesis. This conclusion was reinforced by the results of REST-focused research on neural development from non-human animal models, giving us important proof that REST deprivation negatively affects brain development. A germline knockout (KO) of the REST gene was found to be fatal for mice (Chen et al. 1998). Mice with all REST coding exons ablation in NPCs eventually acquire smaller brains due to apoptosis and DNA damage. This suggests that REST is necessary to sustain proper cell division and differentiation during brain development (Nechiporuk et al. 2016). However, another study demonstrated no aberrant brain development after brain-specific deletion of REST via Cre-loxP recombination, as proved in vivo (Aoki 2018). This study demonstratedconflicting findings. The present study showed a significant role of REST signaling in neurodevelopment in humans.

## Conclusion

REST expression is seen in hiPSCs, immature neurons, and mature neurons derived from hiPSCs. The inhibition of REST in hiPSCs, NSCs and neuronal precursor cells with X5050 significantly reduced REST levels. Reduced REST levels in NSCs led to significant downregulation of essential proteins involved in neurogenesis, including SOX2, a key regulator of NSC proliferation. Additionally, REST suppression impaired critical signaling pathways, notably the MAPK and WNT pathways, which are vital for neurogenic processes. This study reveals that REST is a crucial regulator of human neurogenesis. REST is a prerequisite to drive neurogenesis as it controls the SOX2 levels during this stage. X5050 reduces NESTIN, β-catenin (CTNNB1), and MAPK3 mRNA levels, indicating effects on neural stem cell identity and WNT/MAPK signaling. These findings underscore the function of REST in regulating neurogenic pathways and offer novel perspectives on its potential as a target for therapy of neurodevelopmental diseases.

## Electronic supplementary material

Below is the link to the electronic supplementary material.

Supplementary Material 1

Supplementary Material 2

## Data Availability

The corresponding author can provide the datasets created and/or analysed during the current work upon reasonable request.

## Abbreviations

REST: RE-1 silencing transcription factor
NRSF: Neuron-restrictive silencing factor
RE-1: Repressor Element 1
NSC(s): Neural stem cell(s)
hiPSC(s): Human induced pluripotent stem cells
NIM: Neural Induction Medium
NPCs: Neuroectodermal progenitor cells
PFA: Paraformaldehyde
BSA: Bovine serum albumin
PBS: Phosphate-buffered saline
RIPA: Radioimmunoprecipitation assay
BCA: Bicinchoninic acid
PVDF: Polyvinylidene fluoride
LC-MS: Liquid chromatography-mass spectrometry
MS/MS: Tandem mass spectrometry
C18: Octadecyltrichlorosilane
SOX2: SRY-box transcription factor 2
HDACs: Histone deacetylases
GluN2B: NMDA receptor subunit 2B
GluN2A: NMDA receptor subunit 2 A
NMDAR: N-Methyl-D-Aspartate receptor
CoREST: Corepressor of REST
SIN3: Switch-independent 3
MECP2: Methyl CpG binding protein 2
LSD1: Lysine-specific demethylase 1
X5050: REST inhibitor
TF(s): Transcription factor(s)
BDNF: Brain-derived neurotrophic factor
HEK293: Human embryonic kidney 293 cells
AD: Alzheimer’s disease
PD: Parkinson’s disease
HD: Huntington’s disease
MKI67: Marker of proliferation Ki-67
YAP1: Yes-associated protein 1
KAT7: Histone acetyltransferase 7
KDM1A: Lysine-specific demethylase 1 A
NES: Nestin
CTNNB1: Catenin beta 1
BYSL: Bycine small ribosomal subunit
HNRNPU: Heterogeneous nuclear ribonucleoprotein U
HMGB2: High mobility group box 2
TIAL1: TIA1 cytotoxic granule-associated RNA binding protein 1
MAPK: Mitogen-activated protein kinase
WNT: Wingless/Integrated signaling pathway
cAMP: Cyclic adenosine monophosphate

## Declarations

### Declarations

Nil.
