# Supplementary material for: Pharmacological inhibition of RE1 silencing transcription factor disrupts SOX2 expression and neurogenesis in human induced pluripotent stem cells derived neuronal models
Source: Metab Brain Dis. 2025 Nov 18;40(8):318. doi: 10.1007/s11011-025-01744-1 (PMC12627181; doi:10.1007/s11011-025-01744-1)

WESTERN BLOT IMAGES

Fig 2

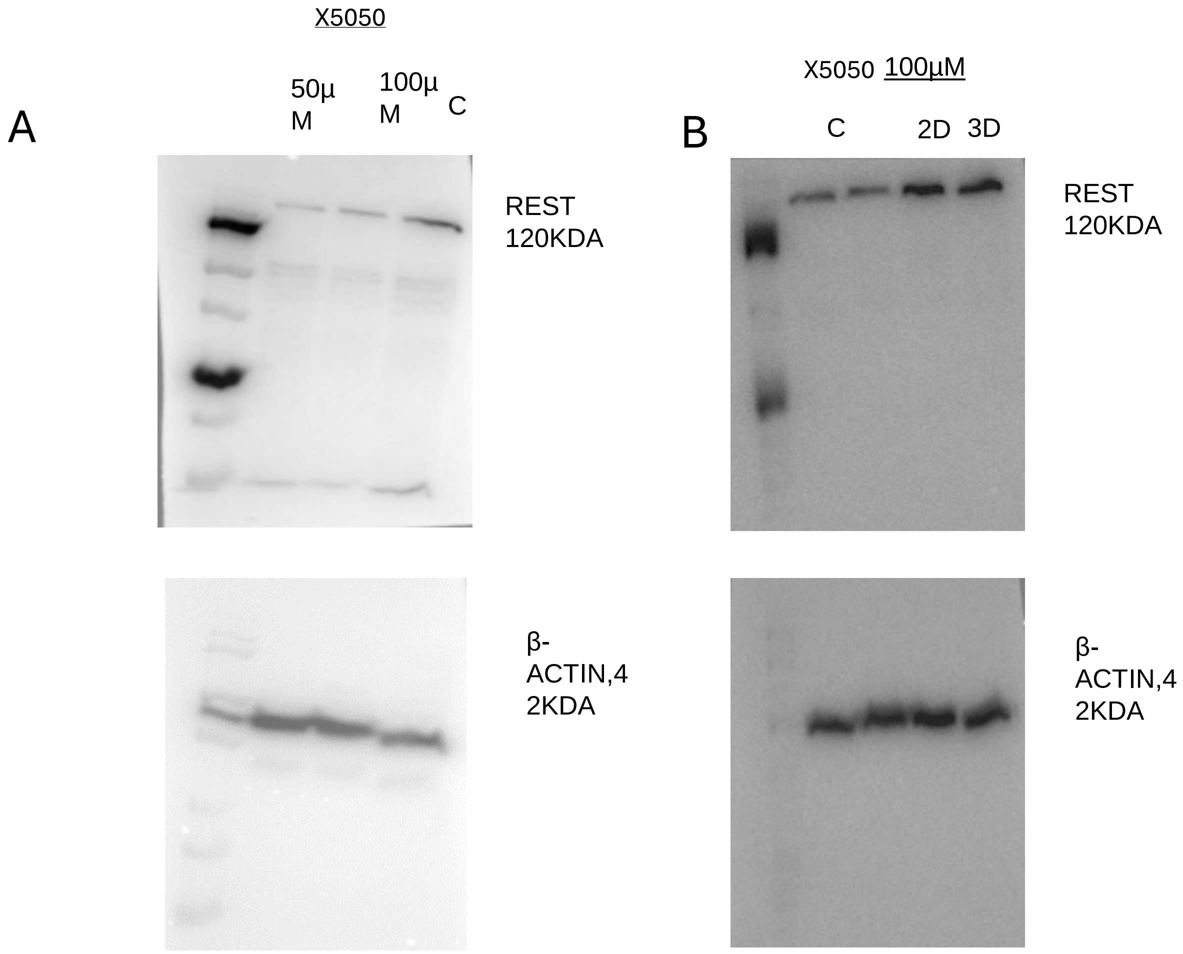

iPSC Cells were treated with X5050 (50 µM, 100 µM) for 24 h and blotted against REST protein, iPSC Cells were treated with X5050 (100µM) for 48 h, 72 h and blotted against REST protein

Western blot analysis was performed to assess the expression of REST and β-actin in NSCs treated with 100 µM X5050 for 24 hours. REST was detected at 120 kDa, while β-actin appeared at 42 kDa.

REST 120 kDa

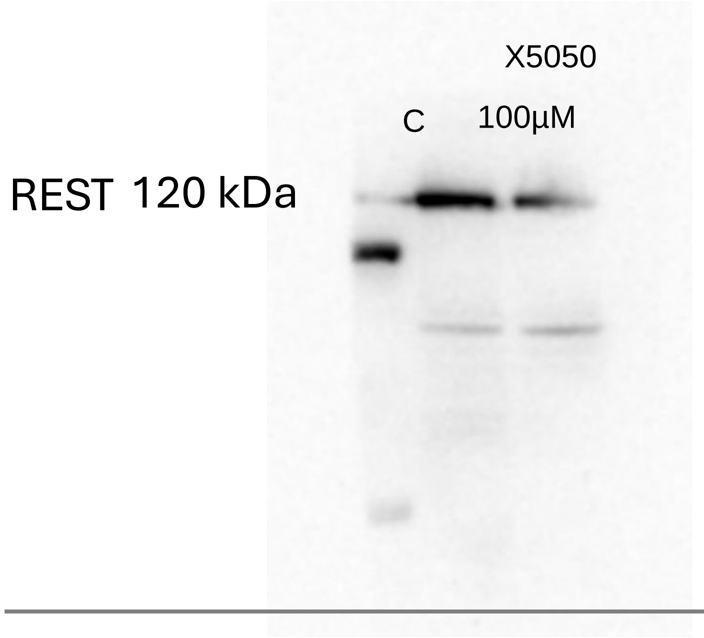

β-actin 42 kDa

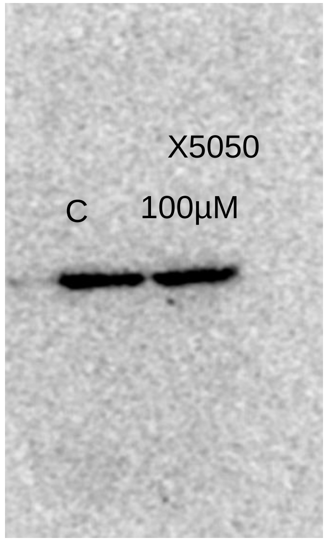

REST 120 kDa

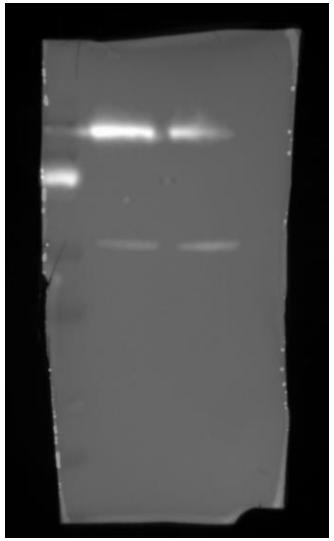

β-actin 42 kDa

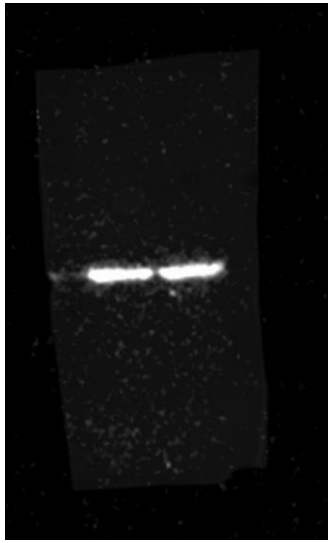

Western blot analysis was performed to assess the expression of REST and β-actin in Neural progenitor cells (Day 25) treated with X5050 at 100 µM. REST was detected at 120 kDa, while β-actin appeared at 42 kDa.

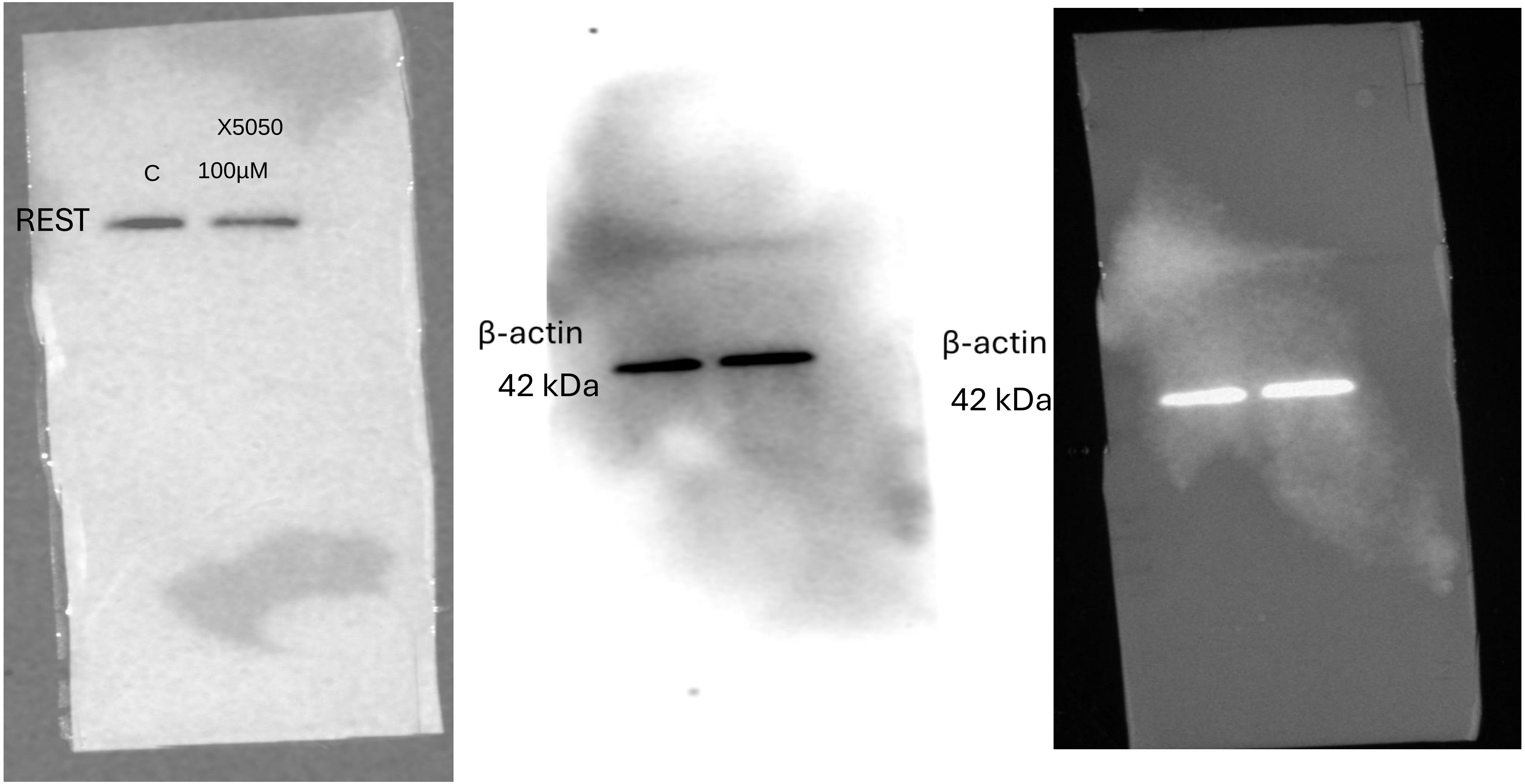

Western blot analysis of SOX2 and β-actin in (a) NSCs treated with X5050 (100 µM) for 24 hours

SOX2 35 kDa

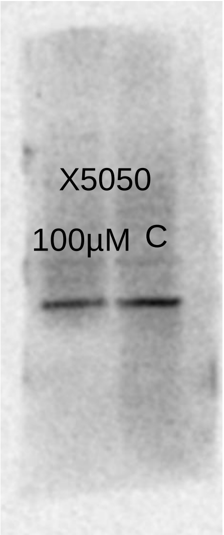

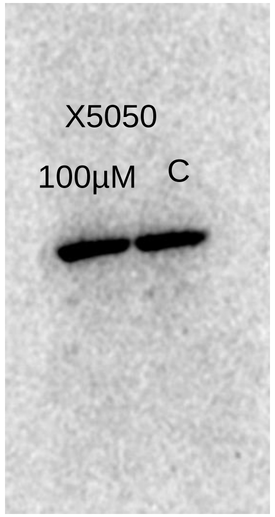

β-actin 42 kDa

SOX2 35 kDa

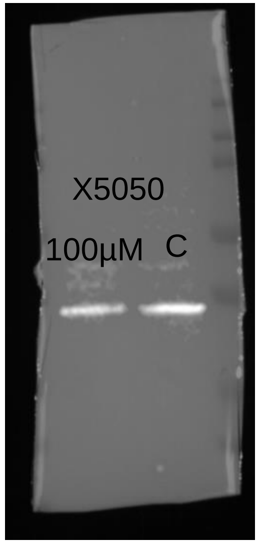

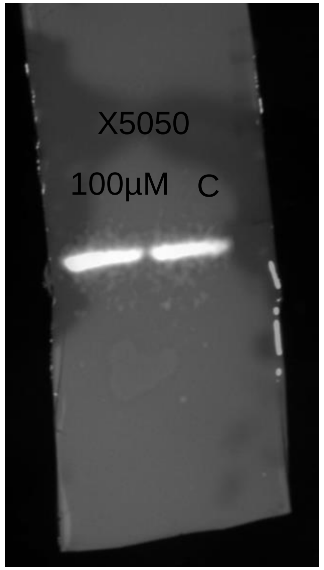

β-actin 42 kDa

Western blot analysis of SOX2 and β-actin in NSCs treated with X5050 (100 µM) every 24 hours from Day 3 to Day 9, with Western blotting performed on Day 9

SOX2 35 kDa

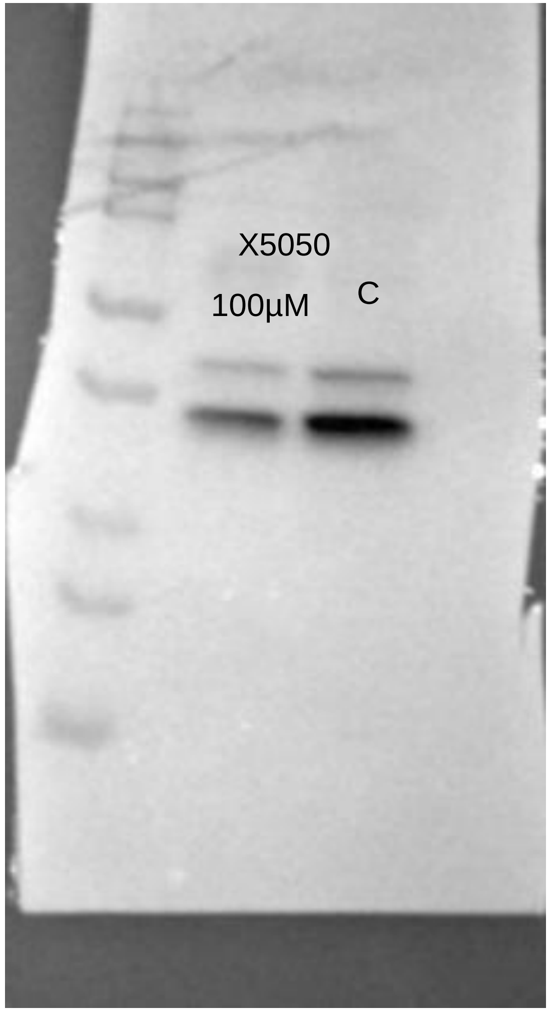

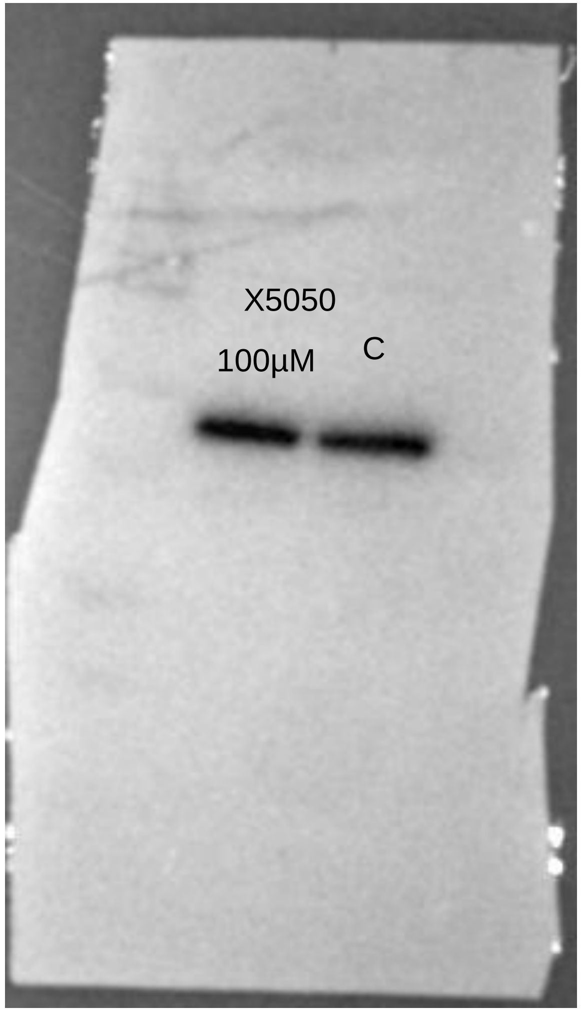

β-actin 42 kDa

Western blot analysis of SOX2 and β-actin in NPCs at Day 25 with and without X5050 treatment

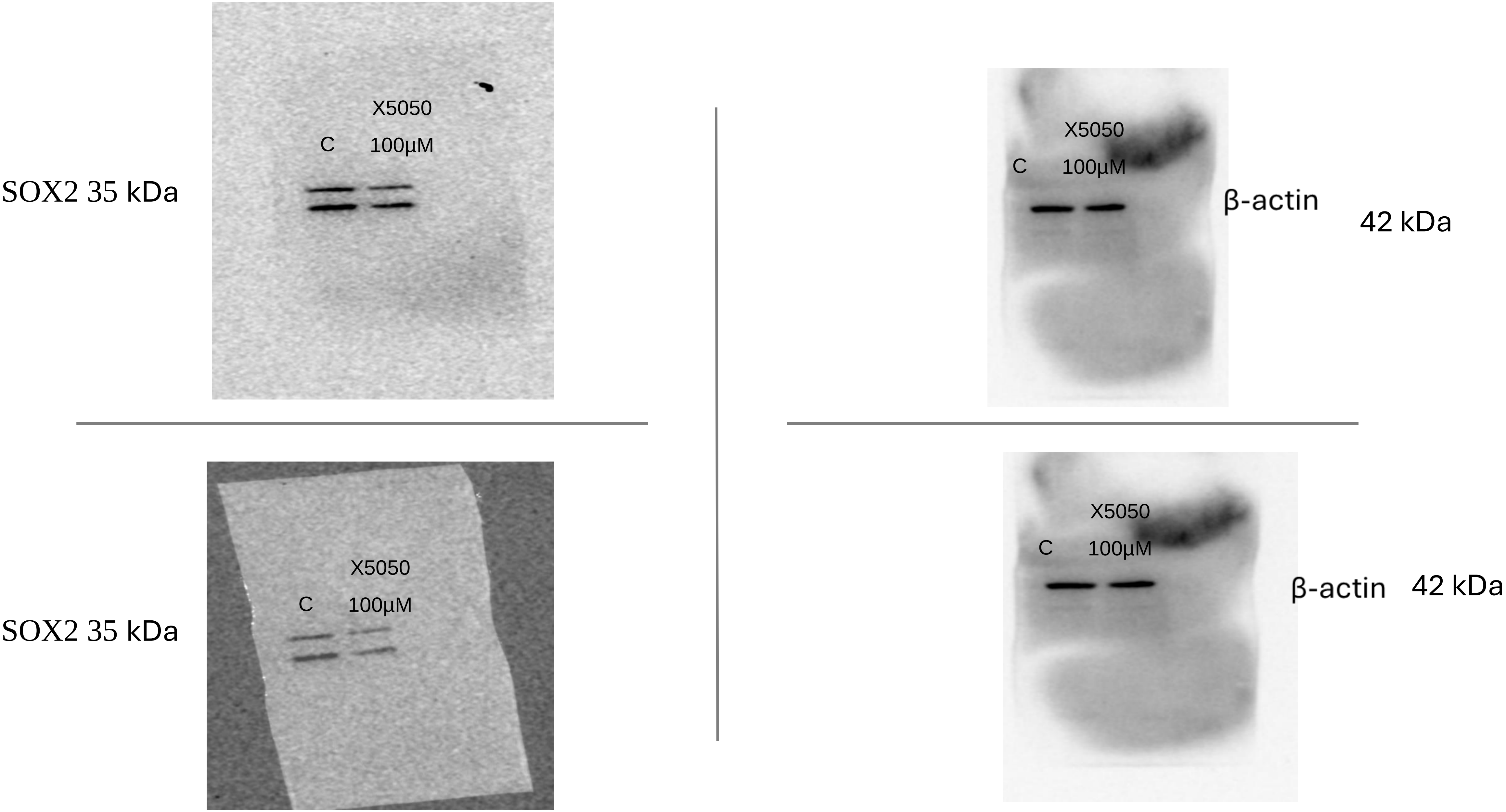

Supplement: Supplementary file 1 — Supplementary Material 1 [file 11011_2025_1744_MOESM1_ESM.pdf]
